# Interaction of Chrysin and Its Main Conjugated Metabolites Chrysin-7-Sulfate and Chrysin-7-Glucuronide with Serum Albumin

**DOI:** 10.3390/ijms19124073

**Published:** 2018-12-17

**Authors:** Violetta Mohos, Eszter Fliszár-Nyúl, Gabriella Schilli, Csaba Hetényi, Beáta Lemli, Sándor Kunsági-Máté, Balázs Bognár, Miklós Poór

**Affiliations:** 1 Department of Pharmacology, University of Pécs, Faculty of Pharmacy, Szigeti út 12, H-7624 Pécs, Hungary; mohos.violetta@gytk.pte.hu (V.M.); eszter.nyul@aok.pte.hu (E.F.-N.); 2 János Szentágothai Research Center, University of Pécs, Ifjúság útja 20, H-7624 Pécs, Hungary; lemli.beata@gytk.pte.hu (B.L.); kunsagi-mate.sandor@gytk.pte.hu (S.K.-M.); 3 Department of Pharmacology and Pharmacotherapy, University of Pécs, Medical School, Szigeti út 12, H-7624 Pécs, Hungary; scgpecs@gmail.com (G.S.); csabahete@yahoo.com (C.H.); 4 Department of Pharmaceutical Chemistry, University of Pécs, Faculty of Pharmacy, Rókus utca 2, H-7624 Pécs, Hungary; 5 Department of Organic and Pharmacological Chemistry, University of Pécs, Medical School, Honvéd utca 1, H-7624 Pécs, Hungary; bognar.balazs83@gmail.com

**Keywords:** chrysin, chrysin-7-sulfate, chrysin-7-glucuronide, serum albumin, fluorescence spectroscopy, albumin–ligand complexes

## Abstract

Chrysin (5,7-dihydroxyflavone) is a flavonoid aglycone, which is found in nature and in several dietary supplements. During the biotransformation of chrysin, its conjugated metabolites chrysin-7-sulfate (C7S) and chrysin-7-glucuronide (C7G) are formed. Despite the fact that these conjugates appear in the circulation at much higher concentrations than chrysin, their interactions with serum albumin have not been reported. In this study, the complex formation of chrysin, C7S, and C7G with human (HSA) and bovine (BSA) serum albumins was investigated employing fluorescence spectroscopic, ultrafiltration, and modeling studies. Our major observations/conclusions are as follows: (1) Compared to chrysin, C7S binds with a threefold higher affinity to HSA, while C7G binds with a threefold lower affinity; (2) the albumin-binding of chrysin, C7S, and C7G did not show any large species differences regarding HSA and BSA; (3) tested flavonoids likely occupy Sudlow’s Site I in HSA; (4) C7S causes significant displacement of Sudlow’s Site I ligands, exerting an even stronger displacing ability than the parent compound chrysin. Considering the above-listed observations, the high intake of chrysin (e.g., through the consumption of dietary supplements with high chrysin contents) may interfere with the albumin-binding of several drugs, mainly due to the strong interaction of C7S with HSA.

## 1. Introduction

Flavonoids are polyphenolic compounds, which are widely distributed in the plant kingdom. Flavonoids can exert several beneficial effects in the body [1]; however, they can also interact with serum albumin [2,3], biotransformation enzymes [4], and transport proteins [5], leading to their ability to affect the pharmacokinetics of drugs [6]. Nowadays, many dietary supplements contain extremely high doses of flavonoids (ranging from several hundreds to thousands of milligrams), resulting in their unusually high appearance in the blood (and likely in tissues) [7]. After oral consumption/administration, flavonoids commonly undergo biotransformation; therefore, their naturally occurring structure may not have significant oral bioavailability [8]. Therefore, mainly the metabolites appear at high concentrations in the circulation.

**Figure 1:**
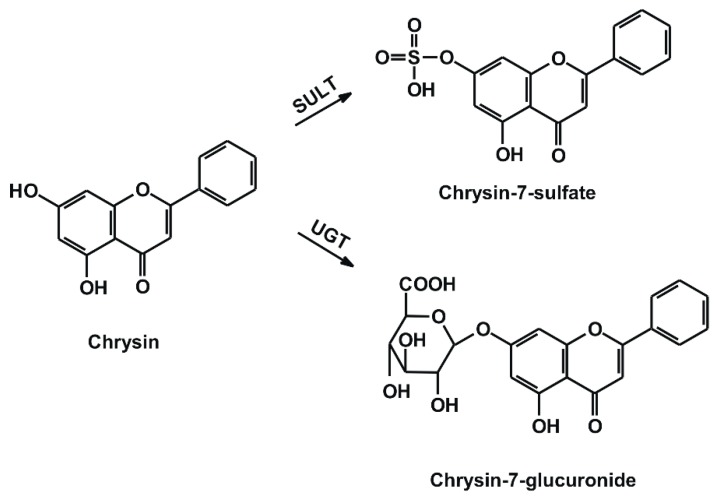
Chemical structures of chrysin, chrysin-7-sulfate, and chrysin-7-glucuronide (SULT: sulfotransferase; UGT: uridine 5′-diphospho-glucuronosyltransferase).

Chrysin (5,7-dihydroxyflavone; Figure 1) is a flavonoid aglycone, which occurs in propolis, honey, flowers, mushrooms, and fruits [9,10,11]. Based on in vitro studies, chrysin inhibits the aromatase enzyme (which converts androstenedione to estrone and testosterone to estradiol) [12,13]; therefore, chrysin is widely used as a dietary supplement to maintain testosterone levels. Ciftci et al. [14] suggest that the oral consumption of chrysin (50 mg/kg per day) exerts positive reproductive effects and might be useful for the treatment of male infertility. As previous studies described, chrysin exerts a neuroprotective effect and alleviates neuro-inflammation and depression in mice [15,16]. In diabetic rats, orally administered chrysin normalized glucose and insulin levels [17]. Furthermore, chrysin showed anti-inflammatory [18,19] and antioxidant effects in rats [20]. Despite the absence of human clinical evidences, chrysin-containing dietary supplements are advertised to treat anxiety, inflammation, gout, erectile dysfunction, baldness, and even cancer (see Supplementary Material). The oral bioavailability of chrysin is low, due to its poor aqueous solubility and significant presystemic elimination in enterocytes and hepatocytes [7,21]. As a result of its biotransformation, conjugated metabolites are formed (Figure 1): The two dominant products are chrysin-7-sulfate (C7S) and chrysin-7-glucuronide (C7G) in humans and in mice [7,22]. After the oral administration of 20 mg/kg chrysin to mice, the C_max_ of chrysin was only 10 nmol/L, while 160 and 130 nmol/L peak plasma concentrations of C7S and C7G were quantified, respectively [22]. In another study, a 400 mg dose of chrysin was administered orally to healthy human volunteers, after which C7S reached approximately 30-fold higher AUC_0–∞_ values compared with chrysin (420–4220 ng·mL^−1^·h vs. 3–16 ng·mL^−1^·h, respectively) [7]. Based on previous studies, chrysin is a potent inhibitor of some biotransformation enzymes (e.g., CYP3A4, CYP2C9, and xanthine oxidase) and is also able to affect drug transporters (e.g., P-glycoprotein) [5,23,24].

Human serum albumin (HSA) is the most abundant plasma protein [25,26]. Drugs and other xenobiotics commonly occupy one of the two major binding sites of HSA, namely Sudlow’s Site I and Sudlow’s Site II. Site I is an apolar cavity in Subdomain IIA [26]. It contains the only tryptophan residue of HSA (Trp-214), and specific ligands include warfarin and furosemide. Site II is located in Subdomain IIIA; e.g., naproxen and ibuprofen occupy this binding site with high affinity [26]. Displacement of a drug from HSA leads to the elevated free (not protein-bound) plasma concentration of the drug, which can affect its tissue uptake and/or elimination [25]. Chrysin binds to HSA with high affinity (*K* is in the 10^5^–10^6^ L/mol range), occupying Site I or Site II as its high-affinity binding site on HSA [2,27,28]. However, based on our current knowledge, the interactions of C7S and C7G with serum albumin have not been reported, despite the fact that these are the dominant metabolites in the circulation.

In this study, the complex formation of chrysin, C7S, and C7G with HSA was investigated. Binding constants of formed flavonoid–albumin complexes were determined based on fluorescence quenching. Binding sites of chrysin, C7S, and C7G in HSA were examined using site markers of Sudlow’s Site I (warfarin and ochratoxin A) and Sudlow’s Site II (naproxen) by steady-state fluorescence spectroscopy, fluorescence anisotropy, ultrafiltration, and molecular modeling. This study demonstrates that both C7S and C7G form stable complexes with HSA. C7S binds with even greater affinity to HSA compared to chrysin, and it exerts stronger displacing ability vs. Sudlow’s Site I ligands than the parent compound. Considering the above-listed observations, a high intake of chrysin may interfere with the albumin-binding of some drugs, mainly due to the strong interaction of C7S with HSA.

## 2. Results

### 2.1. Interaction of Chrysin, C7S, and C7G with Human and Bovine Serum Albumins

**Figure 2:**
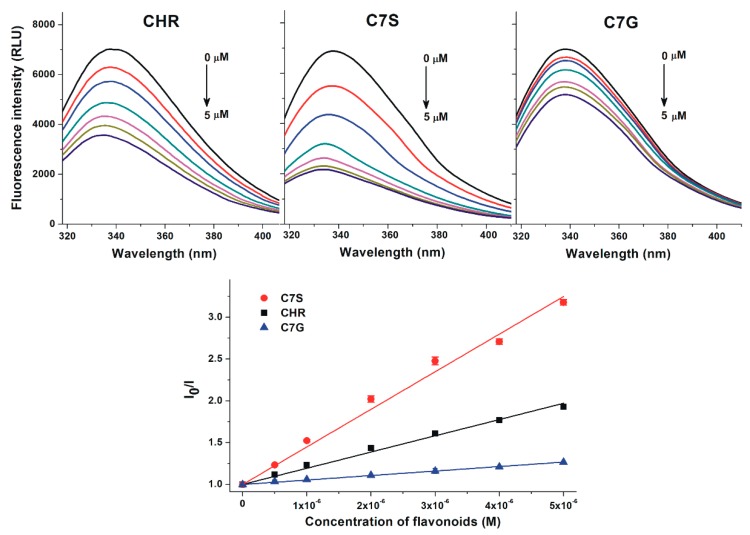
Top graphs: Fluorescence quenching effects of chrysin (CHR; left), chrysin-7-sulfate (C7S; middle), and chrysin-7-glucuronide (C7G; right) on HSA (2 µmol/L) in PBS (pH 7.4; λ_ex_ = 295 nm, λ_em_ = 340 nm). Bottom graph: Stern–Volmer plots of flavonoid–HSA complexes.

**Figure 3:**
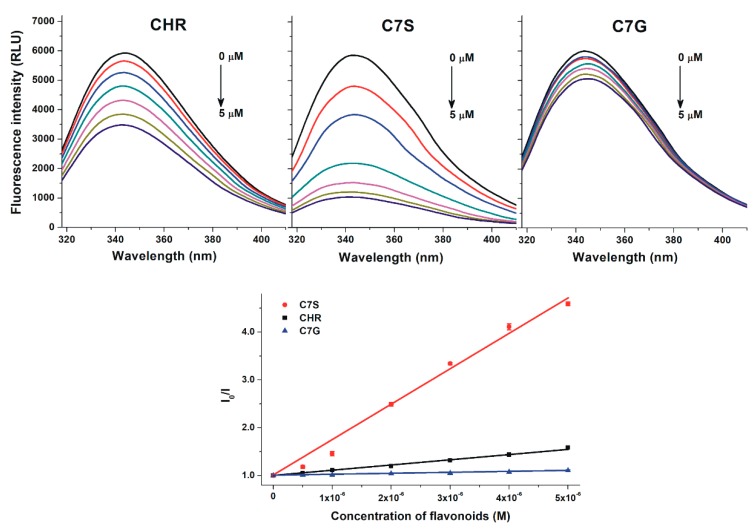
Top graphs: Fluorescence quenching effects of chrysin (CHR; left), chrysin-7-sulfate (C7S; middle), and chrysin-7-glucuronide (C7G; right) on BSA (2 µmol/L) in PBS (pH 7.4; λ_ex_ = 295 nm, λ_em_ = 340 nm). Bottom graph: Stern–Volmer plots of flavonoid–BSA complexes.

To investigate the binding affinity of chrysin, C7S, and C7G toward HSA and bovine serum albumin (BSA), fluorescence quenching experiments were performed. BSA is commonly applied as a model protein to examine albumin–ligand interactions (because it is cheaper and structurally similar to HSA) [29]. In a concentration-dependent fashion, chrysin and its metabolites induced a significant decrease in the fluorescence emission signal of HSA at 340 nm (Figure 2, top; λ_ex_ = 295 nm). C7G caused weaker, and C7S produced stronger, quenching effects on HSA than the parent compound. Stern–Volmer plots of flavonoid–HSA interactions show a good linearity (Figure 2, bottom). Chrysin and its metabolites induced very similar quenching effects on BSA compared to HSA (Figure 3). Stern–Volmer quenching constants (*Ksv*) and binding constants (*K*) were calculated based on the graphical application of the Stern–Volmer equation and evaluated by Hyperquad2006 software, respectively. Table 1 demonstrates the decimal logarithmic values of *Ksv* and *K* for flavonoid–albumin complexes. Both albumins formed the most stable complexes with C7S followed by chrysin and C7G. The stability of C7S–HSA and C7G–HSA complexes are approximately threefold higher and threefold lower vs. the chrysin–HSA complex, respectively. Affinities of the individual flavonoid–HSA and flavonoid–BSA complexes are similar; however, chrysin and C7G bind to HSA stronger than they do to BSA, while C7S forms a more stable complex with BSA (Table 1).

### 2.2. Effects of Flavonoids on Warfarin–HSA and Naproxen–HSA Interactions Based on Ultrafiltration Studies

**Figure 4:**
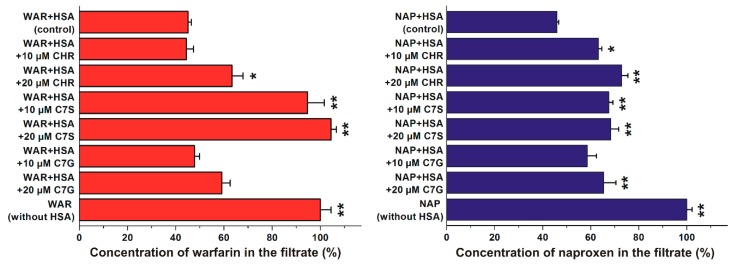
Concentrations of warfarin (left) and naproxen (right) in the filtrate. Left: Before ultrafiltration, samples contained 1 µmol/L warfarin (WAR) with 5 µmol/L HSA in the absence and presence of 10 or 20 μM flavonoid concentrations in PBS (pH 7.4). Right: Before ultrafiltration, samples contained 1 µmol/L naproxen (NAP) with 1.7 µmol/L HSA in the absence and presence of 10 or 20 μM flavonoid concentrations in PBS (pH 7.4). (* *p* < 0.05, * *p < 0.01; CHR: chrysin; C7S: chrysin-7-sulfate; C7G: chrysin-7 glucuronide).

In the following experiments, the displacing ability of chrysin, C7S, and C7G vs. the Site I ligand warfarin and the Site II ligand naproxen was evaluated based on ultrafiltration experiments. Interestingly, chrysin and its metabolites influenced the filtered fractions of both warfarin and naproxen. Under the applied conditions, C7G did not significantly change the concentration of filtered warfarin, while only the higher concentration of chrysin (20 μM) induced a statistically significant (albeit slight) increase of warfarin in the filtrate (Figure 4, left). However, even a 10 μM concentration of C7S caused a remarkable increase in warfarin in the filtrate. Furthermore, both concentrations of chrysin and C7S induced a statistically significant elevation of naproxen concentrations in the filtrate, while only a higher concentration of C7G produced a significant effect (Figure 4, right).

### 2.3. Effects of Chrysin and Its Metabolites on the Albumin-Binding of Warfarin Based on Fluorescence Spectroscopic Studies

**Figure 5:**
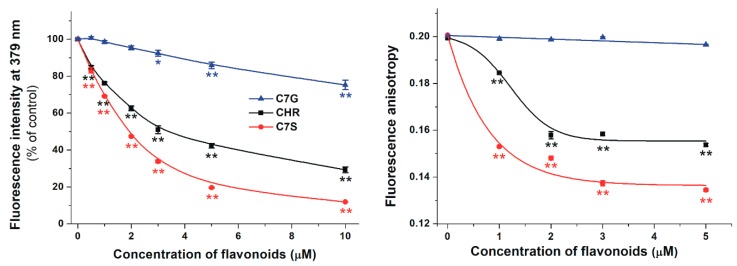
Left: A concentration-dependent decrease in the fluorescence emission intensity of the warfarin–HSA complex (1.0 and 3.5 µmol/L of warfarin and HSA, respectively) in the presence of increasing flavonoid concentrations (0.0, 0.5, 1.0, 2.0, 3.0, 5.0, and 10 µmol/L). Right: Fluorescence anisotropy values of the warfarin–HSA complex (1.0 and 2.0 µmol/L of warfarin and HSA, respectively) in the presence of increasing concentrations of chrysin, C7S, or C7G (0.0, 1.0, 2.0, 3.0, and 5.0 µmol/L). (λ_ex_ = 317 nm, λ_em_ = 379 nm; * *p* < 0.05, ** *p* < 0.01; CHR: chrysin; C7S: chrysin-7-sulfate; C7G: chrysin-7 glucuronide).

In order to test the displacement of the Sudlow’s Site I ligand warfarin from HSA by flavonoids, increasing concentrations of chrysin and its metabolites were added to the warfarin–HSA complex. Displacement of warfarin from HSA results in a strong decrease in its fluorescence [30,31]. Our results demonstrate that each flavonoid significantly decreased the emission signal of warfarin at 379 nm (Figure 5, left): C7S induced the strongest decrease, followed by chrysin, while C7G showed only a slight (but statistically significant) effect.

In another model, fluorescence anisotropy was employed to test the effects of chrysin, C7S, and C7G on the albumin-binding of warfarin. Anisotropy gives information regarding the rotational freedom of a molecule. Our results demonstrate that C7G failed to affect the fluorescence anisotropy of warfarin, while chrysin and mainly C7S induced a strong decrease in anisotropy values (Figure 5, right).

### 2.4. Effects of Chrysin and Its Metabolites on the Albumin-Binding of Ochratoxin A

**Figure 6:**
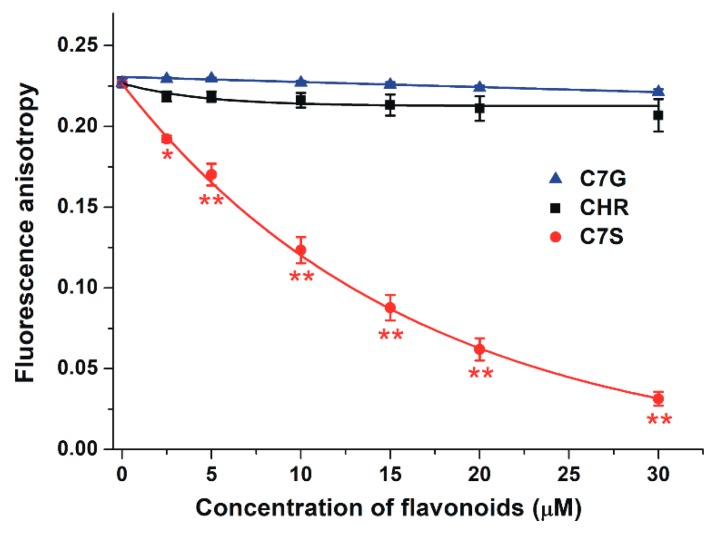
Fluorescence anisotropy of the ochratoxin A–HSA complex (1.0 and 1.5 µmol/L of ochratoxin A and HSA, respectively) in the presence of increasing concentrations of flavonoids (0.0, 2.5, 5.0, 10, 15, 20, and 30 µmol/L) in PBS (pH 7.4; λ_ex_ = 393 nm; λ_em_ = 446 nm; * *p* < 0.05, ** *p* < 0.01; CHR: chrysin; C7S: chrysin-7-sulfate; C7G: chrysin-7 glucuronide).

The mycotoxin ochratoxin A binds to HSA with very high affinity, occupying Sudlow’s Site I as a binding site [32,33]. Therefore, to confirm the involvement of Site I as the binding site of flavonoids tested, the displacement of ochratoxin A from HSA by chrysin, C7S, and C7G was examined by fluorescence anisotropy. Even high concentrations (2.5–30 μM vs. 1 μM ochratoxin A) of C7G and chrysin failed to affect the anisotropy of ochratoxin A; however, C7S induced a concentration-dependent decrease in anisotropy values (Figure 6).

### 2.5. Modeling Studies

**Figure 7:**
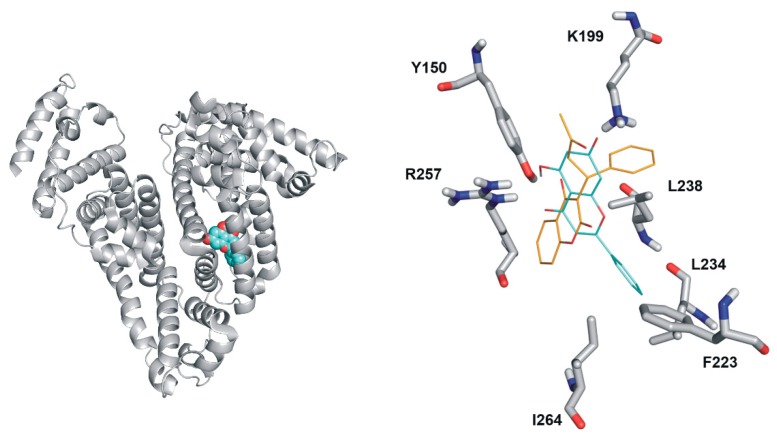
Left: Chrysin (cyan spheres) occupies Sudlow’s Site I in HSA (represented with grey cartoon). Right: Rank1 binding positions of chrysin (cyan lines) and warfarin (orange lines; reference structure) in the HSA molecule.

**Figure 8:**
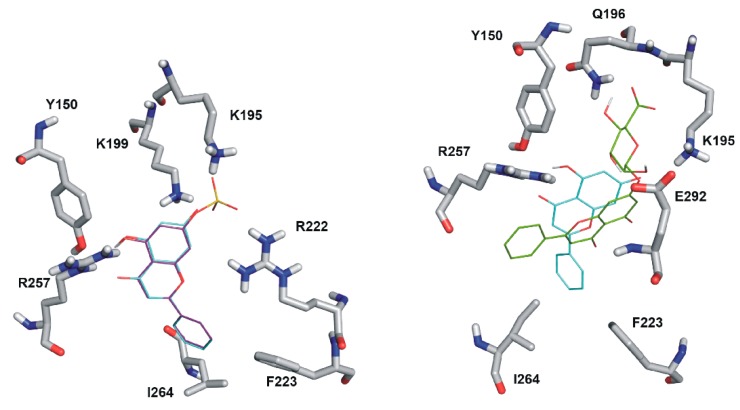
Left: Rank1 binding position of C7S (purple lines) vs. chrysin (cyan lines). Right: Rank1 binding position of C7G (green lines) compared to chrysin (cyan lines).

The binding positions of chrysin, C7S, and C7G in Sudlow’s Site I were investigated employing focused docking calculations with Autodock 4. Ranking of binding positions was performed by the binding energy calculated with the AutoDock 4 scoring function. Docking results show that the binding site of chrysin on HSA significantly overlaps with the binding site of the Sudlow’s Site I ligand warfarin (Figure 7). The first rank positions of chrysin and C7S show large similarities (Figure 8, left). The chrysin–HSA interaction is stabilized by the salt bridge with K199 and by hydrogen bonds with R257 and Y150. C7S occupies Site I in a very similar position than chrysin, but the formed complex is further stabilized by salt bridges with K195 and R222. The binding position of C7G is different (Figure 7, left) compared to chrysin and C7S. The position of glucuronic acid moiety is fixed by hydrogen bonds with K195, Q196, and E292.

## 3. Discussion

Chrysin, C7S, and C7G induced the significant decrease in the fluorescence signal of HSA (Figure 2, top), suggesting the formation of flavonoid–HSA complexes. Since emission signals were corrected with absorbance values of flavonoids at the excitation and emission wavelengths used (see in 4.2), we can exclude that the decrease in fluorescence resulting from the inner-filter effect. Using 295 nm as an excitation wavelength, the emitted light of HSA was exerted mainly by its only tryptophan moiety (Trp-214), which is located in Sudlow’s Site I [26,32,33]. Because flavonoids caused the significant quenching of Trp-214 even at a relatively low concentration (0.5 μmol/L), it is reasonable to hypothesize that the binding site of flavonoids needs to be located close to the tryptophan residue (e.g., in Site I). Both the good linearity of Stern–Volmer plots (Figure 2, bottom) and the evaluation of data with the Hyperquad software suggest the 1:1 stoichiometry of complex formation. *K_SV_* and *K* values showed good correlations (Table 1). Binding constant of the chrysin–HSA complex is in good agreement with the previously reported values [2,27,28]. Sometimes significant species differences in albumin-binding can be noticed [29,34]; however, the stability of the formed HSA and BSA complexes of chrysin (and chrysin conjugates) did not show large differences, as has also been described regarding the basic flavone structure by Xiao et al. [2]. Like previous investigations with quercetin and its sulfate metabolites [30,35,36,37], our results highlight that sulfate metabolites of flavonoids are able to bind to albumin with similar or even higher affinities than the aglycones. Considering these observations, it is reasonable to hypothesize that sulfate metabolites may also exert similar or higher effects on other proteins (e.g., biotransformation enzymes or transporters) than flavonoid aglycones, as has been reported regarding the interactions of quercetin-3’-sulfate with CYP2C9, CYP3A4, OAT1, or GLUT [30,38,39].

In order to test the binding sites of flavonoids in HSA as well as to investigate their displacing ability vs. drugs, site markers were applied. Since previous investigations suggest the possible involvement of Site I or Site II regarding chrysin–HSA interaction [2,28], the displacing effects of chrysin, C7S, and C7G were evaluated vs. warfarin (Site I) and naproxen (Site II) based on ultrafiltration studies. Because albumin is a large macromolecule (66.5 kDa), HSA and HSA-bound ligand molecules cannot pass through the filter unit with a 30 kDa molecular weight cut-off value. Therefore, albumin significantly decreases the concentrations of the site markers in the filtrate (Figure 4). In both cases (warfarin and naproxen), the same concentrations of the site markers (1 μmol/L both) were applied in the presence of HSA concentrations that induce an approximately 60% decrease in the site marker concentrations in the filtrate. Thus, the flavonoid-induced increase in filtered site marker concentrations suggests their displacement from HSA. C7G did not cause significant changes and chrysin induced slight (but statistically significant) interaction only at the higher concentration applied (20 μmol/L); however, C7S inflicted remarkable effects at both concentrations, resulting in practically the complete displacement of bound warfarin molecules from HSA (Figure 4, left). Chrysin, C7S, and C7G induced statistically significant (but only slight) displacement of the Site II marker naproxen (Figure 4, right). Based on these observations, it is reasonable to hypothesize that the high-affinity binding site of C7S is located in Site I (subdomain IIA), while the lower displacing ability of chrysin and C7G likely resulted from their lower affinity toward HSA. This hypothesis is also supported by the strong quenching effects of flavonoids on HSA, because the Trp-214 moiety is located in Subdomain IIA. Furthermore, chrysin and its conjugates may allosterically influence the albumin-binding of the Site II ligand naproxen as well. 

To confirm our hypothesis that the examined flavonoids occupy Site I in HSA, further experiments were performed. Effects of chrysin, C7S, and C7G on the albumin-binding of warfarin were examined based on steady-state fluorescence spectroscopy and fluorescence anisotropy. The fluorescence emission intensity of HSA-bound warfarin is approximately 20-fold higher compared to free warfarin [30,31]. Therefore, the concentration-dependent reduction of the fluorescence signal of warfarin in the presence of flavonoids (Figure 5, left) suggests the displacement of warfarin from HSA. The displacing ability of flavonoids shows the same order than their binding affinity toward HSA: C7S > chrysin > C7G. In the following experiment, the effect of flavonoids on the fluorescence anisotropy of warfarin was examined. Warfarin, as a small molecule, has a large rotational freedom and consequently a relatively low anisotropy value. However, during its interaction with the HSA, its rotational freedom strongly decreases while its anisotropy value significantly increases. Therefore, the displacement of a small fluorophore from the protein leads to the decrease in its fluorescence anisotropy [31,40]. Anisotropy measurements also suggest the strongest displacement of warfarin by C7S, while a weaker and only a slight impact was noticed regarding chrysin and C7G, respectively (Figure 5, right). Thus, spectroscopic measurements confirmed our results derived from ultrafiltration studies.

To further support these results, the interactions of chrysin, C7S, and C7G were investigated with ochratoxin A. Ochratoxin A occupies Site I in the albumin [32,33], during which it forms extremely stable non-covalent complexes with HSA (log*K* = 7.4–7.6) [29,41]. In these experiments, only C7S was able to significantly decrease the fluorescence anisotropy of ochratoxin A (Figure 6), suggesting that the sulfate metabolite of chrysin can disrupt even this high-affinity interaction. Chrysin and C7G binds with lower affinity to HSA than C7S, which may explain why these flavonoids failed to displace ochratoxin A.

Finally, modeling studies were employed to explore the reason of the different binding affinities of chrysin, C7S, and C7G toward HSA. Our results suggest a similar binding position of chrysin and C7S (Figure 8). The sulfate group of C7S forms further two salt bridges with K195 and R222, which results in more stable complexes of C7S with HSA compared to chrysin. While the different binding position of C7G in Site I may explain its lower binding affinity compared to chrysin and C7S.

Several chrysin-containing dietary supplements are available on the Internet, which contain extremely high doses of chrysin (e.g., 500 mg or even higher dose/formulation unit) (see Supplementary Materials). The consumption of these supplements can result in the appearance of high total chrysin (chrysin and its metabolites) concentrations in the circulation, as has been reported regarding quercetin [42,43]. Since mainly the conjugated metabolites of chrysin appear at high concentrations in the circulation, C7S–HSA and C7G–HSA interactions may have high importance. C7S is the dominant metabolite of chrysin in the human circulation [7]. As it is demonstrated, compared with the parent compound, C7S binds with a higher affinity to HSA and exerts higher displacing ability vs. Sudlow’s Site I ligands. Based on spectroscopic and ultrafiltration studies, C7S has a similar or even higher effect on the albumin-binding of warfarin than quercetin or quercetin-3’-sulfate [30]. Furthermore, the displacement of ochratoxin A by C7S in our experimental model also underlines that C7S is a powerful competitor of Site I ligands. Like chrysin or quercetin-3’-sulfate [23,30,38], C7S is likely able to interact with biotransformation enzymes and transporters that may exacerbate the pharmacokinetic interactions of chrysin. However, to confirm this hypothesis, further investigations are needed.

In summary, interactions of chrysin, C7S, and C7G with human and bovine serum albumins were examined. Binding constants show the formation of stable flavonoid–albumin complexes. C7S binds to HSA with a three-fold higher affinity compared with chrysin, while C7G binds with a threefold lower affinity. Based on site marker experiments and modeling studies, tested compounds occupy Sudlow’s Site I in HSA; however, the binding position of C7G differs from chrysin or C7S. Interestingly, high concentrations of chrysin and its metabolites were also able to influence the albumin-binding of the Sudlow’s Site II ligand naproxen. Considering our observations, the high intake of chrysin (e.g., through the consumption of dietary supplements with high chrysin content) may interfere with the albumin-binding of several drugs, mainly due to the strong interaction of C7S with HSA.

## 4. Materials and Methods

### 4.1. Reagents

Chrysin, racemic warfarin, naproxen, ochratoxin A, human serum albumin (HSA), and bovine serum albumin (BSA) were purchased from Sigma-Aldrich (Waltham, MA, USA). Chyrsin-7-sulfate (C7S) was synthetized based on a method described regarding the sulfation of baicalein [44]. Chrysin-7-glucuronide (C7G) was obtained from Carbosynth (Compton, Berkshire, UK). Chrysin and its metabolites were dissolved in dimethyl sulfoxide (DMSO; Reanal, Budapest, Hungary, spectroscopic grade), and stock solutions (2000 μM each) were stored and protected from light at −20 °C.

### 4.2. Spectroscopic Measurements

Fluorescence spectroscopic measurements were carried out employing a Hitachi F-4500 fluorimeter (Tokyo, Japan). Absorption spectra of flavonoids were recorded using a HALO DB-20 UV-Vis spectrophotometer (Dynamica, London, UK). Spectroscopic measurements were performed in phosphate-buffered saline (pH 7.4; PBS: 8.0 g/L NaCl, 0.2 g/L KCl, 1.81 g/L Na_2_HPO_4_·2H_2_O, and 0.24 g/L KH_2_PO_4_) at room temperature, in the presence of air.

The complex formation of flavonoids with albumin was tested with both HSA and BSA. Binding constants of flavonoid–albumin complexes were determined by fluorescence quenching, during which emission spectra of albumins (2 µmol/L) were recorded in the presence of increasing flavonoid concentrations (0.0, 0.5, 1.0, 2.0, 3.0, 4.0 and 5.0 µmol/L; λ_exc_ = 295 nm). Quenching studies were evaluated based on the graphical application of the Stern–Volmer equation [31,34]. To eliminate the potential inner filter effects of flavonoids, fluorescence intensities were corrected as described [31,34].

Binding constants (*K*) of flavonoid–albumin complexes were also evaluated by non-linear fitting employing the Hyperquad2006 program package (Protonic Software, Leeds, UK) as described elsewhere [34,37]. Binding constants and stoichiometry were quantified based on the model with the lowest standard deviation.

Displacement of warfarin (site marker of Sudlow’s Site I) from HSA was examined based on the previously reported model [30,31]. Increasing concentrations of chrysin, C7S, and C7G (0.0, 0.5, 1.0, 2.0, 3.0, 5.0, and 10 µmol/L) were added to standard concentrations of warfarin and HSA (1.0 and 3.5 µmol/L, respectively). Thereafter, fluorescence emission spectra were recorded using 317 nm as excitation wavelength. 

To confirm further the displacement of warfarin from HSA by chrysin, C7S, and C7G, fluorescence anisotropy measurements were also performed. Increasing concentrations of flavonoids (0.0, 1.0, 2.0, 5.0, and 10 µmol/L) were added to warfarin and HSA (1.0 and 2.0 µmol/L, respectively). Fluorescence anisotropy values were determined (λ_ex_ = 317 nm; λ_em_ = 379 nm) as described [31].

Our previously described fluorescence anisotropy-based model [45] was employed to examine the displacing ability of the test compounds vs. ochratoxin A (another ligand of Sudlow’s Site I). Increasing concentrations of flavonoids (0.0, 2.5, 5.0, 10, 15, 20, and 30 µmol/L) were added to ochratoxin A and HSA (1.0 and 1.5 µmol/L, respectively), after which anisotropy values were determined at 393 and 446 nm excitation and emission wavelengths, respectively (wavelength maxima of albumin-bound ochratoxin A).

### 4.3. Ultrafiltration Experiments

Effects of chrysin, C7S, and C7G on the albumin-binding of Sudow’s Site I (warfarin) and Site II (naproxen) markers were tested by ultrafiltration. In these experiments, Pall Microsep^TM^ Advance Centrifugal Devices (Pall Corporation, Ann Arbor, MI, USA) (from VWR) with a 30 kDa molecular weight cut-off value were employed, as described elsewhere [46]. To examine the displacement of warfarin by flavonoids, samples contained warfarin and HSA (1.0 and 5.0 µmol/L, respectively) in the absence and presence of flavonoids (10 or 20 µmol/L). Furthermore, to test the displacing ability of chrysin and its metabolites vs. naproxen, flavonoids (10 or 20 µmol/L) were added to naproxen and HSA (1.0 and 1.7 µmol/L, respectively). Each sample was prepared in PBS (pH 7.4). Before ultrafiltration, filter units were washed once with 3 mL of water, and twice with 3 mL of PBS (to completely eliminate glycerol from the filters). Thereafter, samples (2.5 mL each) were driven through the filter units by centrifugation at 7500 g and 25 °C for 10 min (fixed angle rotor). Concentrations of warfarin and naproxen in the filtrate were quantified with HPLC (see details in Section 4.4).

### 4.4. HPLC Analyses

The HPLC system used to quantify warfarin and naproxen was equipped with a pump (model 510, Waters, Milford, MA, USA) and an injector (Rheodyne 7125, Rheodyne, Berkeley, CA, USA) with a 20 µL sample loop. Warfarin and naproxen were analyzed with a fluorescence detector (Jasco FP-920, Reanal) and an UV detector (Waters 486), respectively. Data were evaluated using Millennium Chromatography Manager (Waters).

Warfarin was analyzed based on the previously reported method without modifications [46]. Methanol (VWR), acetonitrile (VWR), and sodium phosphate buffer (20 mM, pH 7.0) (25:5:70 *v*/*v*%) were applied in the mobile phase with 1 mL/min flow rate during the isocratic elution. Samples were driven through a guard column (Phenomenex SecurityGuard^TM^ Catridge C18 4.0 × 3.0 mm) linked to an analytical column (Nova-Pak C18 150 × 3.9 mm, 4 μm). Separation and analysis were performed at room temperature, during which warfarin was detected by fluorescence employing 310 and 390 nm as excitation and emission wavelengths, respectively.

Naproxen was quantified based on the previously reported method without modifications [46]. Acetonitrile and sodium acetate buffer (6.9 mM, pH 4.0) (50:50 *v*/*v*%) were applied in the mobile phase with a 1 mL/min flow rate during the isocratic elution. Samples were driven through a guard column (Phenomenex SecurityGuardTM Catridge C18 4.0 × 3.0 mm) linked to an analytical column (Phenomenex Gemini C18 150 × 4.6 mm, 3 µm). Separation and analysis were performed at room temperature, during which naproxen was detected by absorbance at 230 nm.

### 4.5. Modeling Studies

Docking calculations were performed using the AutoDock 4.2 program package [47]. Ligand 3D structures were downloaded from pubchem [48], and the protonation and charge states of ligands were calculated at pH 7.4 by msketch [49]. Energy-minimization of molecules was performed by the semi-empirical quantum chemistry program package, MOPAC. The geometries were optimized at a 0.001 gradient norm and subjected to subsequent force calculations using PM7 parameterization. In all cases, the force constant matrices were positive definite. The apo structure of HSA (PDB code 1a06) was used as a target of docking. The Gasteiger–Marsilli partial charges [50] were added to both ligand and target atoms and a Kollman united atom representation was applied for groups with non-polar bonds. For BD, the grid box was centered on the center of Sudlow’s Site I. A grid map with a box size of 90 × 90 × 90 points and 0.375 Å spacing was calculated by AutoGrid 4. During the focused docking calculations on Sudlow’s Site I, the grid box of 90 × 90 × 90 was centered on 35.000 31.825 37.000. In all calculations, the number of docking runs was set to 10 numbers of energy evaluations and generations were 20 million. Ligand conformations that resulted from the docking runs were ordered by the corresponding calculated ∆G values and clustered using a tolerance of 1.75 Å distance between cluster members. Conformations with the lowest binding energy within a cluster were selected as representatives.

### 4.6. Statistics

Data demonstrate the means (±SEM) from at least three independent experiments. During statistical analyses, the one-way ANOVA test (IBS SPP Statistics Version 21) was applied, and the level of significance was set at *p* < 0.05 and *p* < 0.01.

## Figures and Tables

**Table 1:** Stability of flavonoid–albumin complexes. Decimal logarithmic values (±SEM) of Stern–Volmer quenching constants (*Ksv*, unit: L/mol) and binding constants (*K*, unit: L/mol).

Complex	log*K_SV_*	log*K*
Chrysin–HSA	5.25 ± 0.02	5.41 ± 0.01
Chrysin-7-sulfate–HSA	5.61 ± 0.03	5.88 ± 0.02
Chrysin-7-glucuronide–HSA	4.71 ± 0.03	4.89 ± 0.00
Chrysin–BSA	5.03 ± 0.06	5.20 ± 0.00
Chrysin-7-sulfate–BSA	5.86 ± 0.04	6.20 ± 0.01
Chrysin-7-glucuronide–BSA	4.34 ± 0.03	4.63 ± 0.01

## Supplementary Materials

Supplementary materials can be found at http://www.mdpi.com/1422-0067/19/12/4073/s1.

## Abbreviations

BSA	bovine serum albumin
C7G	chrysin-7-glucuronide
C7S	chrysin-7-sulfate
CHR	chrysin
CYP	cytochrom P450 enzymes
GLUT	glucose transporter
HPLC	high-performance liquid chromatography
HSA	human serum albumin
OAT1	organic anion transporter 1
PBS	phosphate-buffered saline
SULT	sulfotransferase
UGT	uridine 5′-diphospho-glucuronosyltransferase
