# Supplementary material for: Interaction of Chrysin and Its Main Conjugated Metabolites Chrysin-7-Sulfate and Chrysin-7-Glucuronide with Serum Albumin
Source: Int J Mol Sci. 2018 Dec 17;19(12):4073. doi: 10.3390/ijms19124073 (PMC6320863; doi:10.3390/ijms19124073)
Supplement: Supplementary file 1 [file ijms-19-04073-s001.pdf]

Interaction of chrysin and its main conjugated metabolites chrysin-7-sulfate and chrysin-7-glucuronide with serum albumin

SUPPLEMENTARY MATERIALS

Violetta Mohos [1,2], Eszter Fliszár-Nyúl [1], Gabriella Schilli [3], Csaba Hetényi [3], Beáta Lemli [2,4], Sándor Kunsági-Máté [2,4], Balázs Bognár [5], Miklós Poór [1,2,*]

1 Department of Pharmacology, University of Pécs, Faculty of Pharmacy, Szigeti út 12, Pécs H-7624, Hungary; mohos.violetta@gytk.pte.hu (V.M.); eszter.nyul@aok.pte.hu (E.F-N.)
2 János Szentágothai Research Center, University of Pécs, Ifjúság útja 20, Pécs H-7624, Hungary; lemli.beata@gytk.pte.hu (B.L.); kunsagi-mate.sandor@gytk.pte.hu (S.K.)
3 Department of Pharmacology and Pharmacotherapy, University of Pécs, Medical School, Szigeti út 12, Pécs H-7624, Hungary; scgpecs@gmail.com (G.S.); csabahete@yahoo.com (C.H.)
4 Department of Pharmaceutical Chemistry, University of Pécs, Faculty of Pharmacy, Rókus utca 2, Pécs H-7624, Hungary
5 Department of Organic and Pharmacological Chemistry, University of Pécs, Medical School, Honvéd utca 1, Pécs H-7624, Hungary; bognar.balazs83@gmail.com (B.B.)
* Correspondence: poor.miklos@pte.hu; Tel.: +36-536-000 (ext. 34646)

Some examples regarding chrysin-containing dietary supplements which are available in the internet:

First example:

Dose: 500 mg/unit (recommended daily dose: 1 capsule)
Indications: „To support the active man's performance goals while promoting overall wellness."
URL: https://www.ebay.com/itm/Chrysin-500-mg-3X30-Capsules-5-7-dihydroxyflavone/163333170584?_trkparms=aid%3D333200%26algo%3DCOMP.MBE%26ao%3D1%26asc%3D20171012094517%26meid%3D4ba9c42572d3447692b081e429bef39d%26pid%3D100008%26rk%3D1%26rkt%3D11%26sd%3D361888995227%26itm%3D163333170584&_trksid=p2047675.c100008.m2219
Date: November 8, 2018

Second example:

Dose: 500 mg/unit
Indications: „Chrysin may help in homeopathic treatments to fix different problems such as anxiety and depression. Professional athletes may find it useful to their careers since a regular dose may up their testosterone levels. Increased testosterone in men can also help to remedy infertility. A 2014 journal entry from the Polish national public health institute, affirms that flavonoids may cut down certain risks connected to chronic diseases. Flavonoids help to suppress oxidation around the body cells, cutting out the vital supply to free radicals and other harmful carcinogens."
URL: https://www.luckyvitamin.com/p-16909-jarrow-formulas-chrysin-500-mg-30-capsules
Date: November 8, 2018

Third example:

Dose: 750 mg/unit
Indications: „Helps maintain healthy levels of testosterone"
URL: https://www.iherb.com/pr/Life-Extension-Super-Miraforte-with-Standardized-Lignans-120-Veggie-Caps/69067
Date: November 8, 2018

Forth example:

Dose: 900 mg/unit (recommended daily dose: two capsules one hour before training and two capsules at night prior to bed)
Indications: „Based on estrogen levels, the pituitary turns on or off testosterone production. The higher the estrogen levels, the lower the amounts of testosterone will be produced. This is where chrysin comes in! Chrysin basically reduces estrogen levels by cutting down on its conversion from testosterone."
URL: https://www.ebay.com/itm/2-Bottles-PV-Chrysin-UltraMax-Supports-Lean-Muscle-Mass-ZMA-Tribulus-Isoflavones/361888995227?hash=item544243d79b:g:txQAAOSwUKxYiEe0:rk:2:pf:1&frcectupt=true
Date: November 8, 2018

Fifth example:

Dose: 1 kg pure powder
Indications: „- Chrysin Powd-er Extract may have aromatase-inhibitory action.
- Chrysin Powd-er Extract has antitumor activities, can suppress tumor cell multiplication and induce tumor cell apoptosis.
- Chrysin's ability to inhibit the aromatization of androstenedione and testosterone to estrogens has been demonstrated in the laboratory.
- Chrysin Pow-der Extract has the function of antioxidant activity, antiviral activity, antidiabetic action, reducing blood lipids, depressing blood pressure and preventing gene mutation."

URL: https://www.aliexpress.com/item/99-Anticancer-Lowering-blood-lipid-Prevention-of-cardiovascular-and-cerebrovascular-diseases-Chrysin-Powd-er-Extract-1KG/32953048659.html?spm=2114.search0604.3.2.13cf77ee27PZUq&ws_ab_test=searchweb0_0,searchweb201602_3_10065_10068_319_317_10696_5728811_10084_453_454_10083_10618_10304_10307_10820_10821_537_10302_536_5733215_5733315_328_10059_10884_5731015_10887_5733115_100031_5733415_321_322_10103_5733515_5733615,searchweb201603_55,ppcSwitch_0&algo_expid=3529a839-4f65-4487-96db-fe10ead735b1-0&algo_pvid=3529a839-4f65-4487-96db-fe10ead735b1
Date: November 8, 2018
